# 

**Published:** 1881-08

**Authors:** 


Vol. I. AUGUST, 1881. No. 8.

THE

### HOMŒOPATHIC PHYSICIAN,

A MONTHLY JOURNAL OF MEDICAL SCIENCE.

*"Magna est veritas, et prævalebit."*

E. J. LEE, M. D.

EDITOR.

CONTENTS:

(See inside of Cover.)

PHILADELPHIA:

No. 2109 CHESTNUT STREET.

LONDON.

ALFRED HEATH & CO., SOLE AGENTS FOR GREAT BRITAIN,

114 Ebury Street, Eaton Square.

***Yearly Subscription, in advance, $2.00.*** ***Single Numbers, 25 Cents.***

Entered at the Philadelphia Post-Office, as Second-Class Mail Matter.

